# Peritoneal Protein Loss Is Not Associated With Sarcopenia in Peritoneal Dialysis Patients

**DOI:** 10.3389/fmed.2021.653807

**Published:** 2021-07-14

**Authors:** Jun Young Do, A Young Kim, Seok Hui Kang

**Affiliations:** Division of Nephrology, Department of Internal Medicine, College of Medicine, Yeungnam University, Daegu, South Korea

**Keywords:** peritoneal dialysis, sarcopenia, muscle mass, handgrip strength, peritoneal protein loss

## Abstract

**Introduction:** Maintenance of a peritoneal membrane is essential for maintaining long-term peritoneal dialysis (PD). Peritoneal protein loss (PPL) is basically the loss of an essential nutrient, which may lead to malnutrition. We aimed to evaluate the association between PPL and sarcopenia in PD patients.

**Methods:** We conducted a cross-sectional study from September 2017 to November 2020 on all PD patients (*n* = 199). Finally, the patients were divided into tertiles based on the PPL level as follows: low, middle, and high. PPL (mg/day), appendicular lean mass (ALM) using dual-energy X-ray absorptiometry, and handgrip strength (HGS) were evaluated. Sarcopenia was defined using cut-off values from the Asian Working Group for Sarcopenia.

**Results:** The median PPL (interquartile range, interval) in the low, middle, and high tertiles were 4,229 (904, 1,706–5,111), 6,160 (760, 5,118–7,119), and 8,543 (2,284, 7,145–24,406) mg/day, respectively. HGS in the low, middle, and high tertiles was 23.4 ± 9.2, 23.8 ± 8.9, and 23.6 ± 8.3 kg, respectively (*P* = 0.967). The ALM index in the low, middle, and high tertiles was 6.0 ± 1.3, 6.0 ± 1.2, and 6.5 ± 1.1 kg/m^2^, respectively (*P* = 0.061). Multivariate analyses did not reveal significant differences in HGS and ALM index in among tertiles. The proportions of patients with sarcopenia in the low, middle, and high tertiles was 24 (36.4%), 19 (28.4%), and 21 (31.8%), respectively (*P* = 0.612).

**Conclusion:** The present study showed that PPL is not independently associated with muscle mass, strength, and sarcopenia in PD patients.

## Introduction

Peritoneal dialysis (PD) is a modality among renal replacement therapies, and the proportion of end-stage renal disease (ESRD) patients who underwent PD was 4.1% in Korea and 7.5% in U.S.A (1, 2). PD involves removal of uremic molecules and/or water through the peritoneal membrane. Proper characteristics and maintenance of a peritoneal membrane are essential for maintaining long-term PD. Molecular transport via peritoneal membrane is carried out through large pores, small pores, and aquaporins (3, 4). Maintenance of homeostasis for survival in ESRD patients requires proper function and combination of these pores or transporters. A large pore is associated with removal of large sized uremic toxins (middle molecules) and nutrients (albumin).

Previous studies showed that peritoneal protein loss (PPL) in PD patients approximately ranges between 5 and 15 g/day (5–7). Previous studies have focused on the importance of endothelial dysfunction or local/systemic inflammation as underlying pathogenesis for high PPL. Consequently, PD patients with high PPL are at increased risk of cardiovascular disease or mortality. Some studies showed a positive association between PPL and cardiovascular disease or mortality in PD patients (8–12). In contrast to the hazard effects of albumin loss during dialysis, other studies showed that PPL has a favorable effect due to the removal of carbamylated albumin or protein-bound uremic toxin (13, 14). However, PPL is basically the loss of an essential nutrient, which may lead to malnutrition. Previous studies have evaluated the association between PPL and nutritional status in PD patients, but the results were inconsistent (15–18). In most of these studies, laboratory indicators (serum albumin or lean body mass using creatinine kinetics) were used as nutritional markers. Few studies elucidated the associtaion between PPL and sarcopenia (representing muscle strength and muscle mass measurements) through a hard outcome in malnutrition. We therefore aimed to evaluate the association between PPL and sarcopenia in PD patients.

## Methods

### Study Population

We conducted a cross-sectional study from September 2017 to November 2020 on all PD patients, with relevant data, at a tertiary medical center (*n* = 214). Among these, we excluded nine patients with missing data and six patients who were unable to ambulate or had an amputated limb. Therefore, we finally included 199 prevalent PD patients. In our center, handgrip strength (HGS), body composition, and peritoneal equilibration test (PET), including PPL, were annually measured. These measurements were performed on the same day. Cross-sectional data on the measurements obtained at the time of patient assessment were analyzed. If the results of multiple measurements were available during the study period, the most recent samples were used for analyses. This study received ethical approval from the Institutional Review Board of our medical center, which waived the need for informed consent because the patient records and information were anonymized and de-identified prior to analysis. The study was conducted ethically in accordance with the World Medical Association Declaration of Helsinki. Finally, the patients were divided into tertiles based on the PPL level as follows: low, middle, and high.

### Study Variables

Age, sex, presence of diabetes mellitus (DM), dialysis modality, dialysis vintage (months), body mass index (BMI, kg/m^2^), weekly Kt/V_urea_, C-reactive protein (CRP, mg/dL), 4-h dialysate-to-plasma creatinine concentration ratio (DP4_Cr_), urine volume (mL/day), proteinuria (mg/day), edema index, serum calcium (mg/dL), phosphorus (mg/dL), sodium (mEq/L), potassium (mEq/L), albumin (g/dL), and normalized protein equivalent for total nitrogen appearance (nPNA, g/kg/day) levels were collected prior to analyses. DM was defined as a patient-reported history and a medical record of a DM diagnosis or medication. PPL (mg/day) was calculated using concentration of total protein of dialysate x 24-h total drain volume. Weekly Kt/V_urea_ was calculated using 24-h urine and dialysate, as previously published (19). DP4_Cr_ was evaluated using a modified 4.25% PET, and the level was calculated using the creatinine level of the drained dialysate 4 h after injection per the blood creatinine level.

### Assessment of Nutritional Markers

Body composition measurements were evaluated using bioimpedance analysis (BIA) and dual-energy X-ray absorptiometry (DXA). Briefly, the peritoneal dialysate was drained from the abdomen prior to measurement. Each patient was clothed with a light gown, and the bladder was emptied prior to procedures. BIA measurements were performed after a 5-min rest in the erect position. Eight electrodes were placed, two for each foot and two for each hand, with the patient in the erect position. The extracellular and total body water was calculated using regression equation from segmental bioimpedances. The edema index was defined as extracellular water/total body water. DXA (Hologic, Madison, WI, USA) was measured with the patient in the supine position immediately after BIA measurements. The appendicular lean mass (ALM) was calculated using sum of lean masses of both extremities. The index values were defined as the value per squares of height in meters.

The HGS (kg) was measured in all patients after each of three trials with the dominant hand using a digital dynamometer (Takei 5401; Takei Scientific Instruments Co., Ltd, Niigata, Japan). The maximum strength measured over the three trials was recorded. Sarcopenia was defined using cut-off values from the Asian Working Group for Sarcopenia (20). Patients with a low muscle mass (ALM index <7.0 kg/m^2^ for males and <5.4 kg/m^2^ for females using DXA) and low HGS (<26 kg for males and <18 kg for females) were classified as having sarcopenia.

### Statistical Analysis

Data were analyzed using IBM SPSS Statistics version 25 (SPSS Inc., Chicago, IL, USA). Categorical variables were expressed as counts (percentages) and were compared using the chi-square test. The distribution of continuous variables was evaluated using the Kolmogorov-Smirnov test. Continuous variables were expressed as means ± standard deviations for those with normal distribution and medians (interquartile ranges) for those with non-normal distribution (on descriptive univariate analyses). Continuous variables for multivariate analyses were expressed as means ± standard errors (on multivariate analyses). For continuous variables, means were compared using the one-way analysis of variance or Kruskal-Wallis test, followed by a Bonferroni *post-hoc* comparison, and analysis of covariance for multivariate analysis. The correlation between two continuous variables was assessed using Pearson's correlation analysis. Linear regression analysis was performed to assess the independent predictors of nutritional indices. Logistic regression analysis was performed to evaluate the independent predictor of sarcopenia. Multivariate analysis was adjusted for age, sex, the presence of DM, dialysis modality, DP4_Cr_, urine volume, edema index, and serum albumin. The level of statistical significance was set at *P* < 0.05.

## Results

### Baseline Characteristics

The median PPL (interquartile range, interval) in the low, middle, and high tertiles were 4,229 (904, 1,706–5,111), 6,160 (760, 5,118–7,119), and 8,543 (2,284, 7,145–24,406) mg/day, respectively. The mean age in the low, middle, and high tertiles was 54.5 ± 12.7, 55.9 ± 12.0, and 56.3 ± 12.1 years, respectively (Table 1). The proportion of patients with DM or who underwent continuous ambulatory PD (CAPD) was greater in the high tertile group than in the others. DP4_Cr_ and edema index were greater in the high tertile group compared to others. Serum albumin level in the high tertile group was lower than that in the low tertile group. The prevalence of DM as the underlying etiology of end-stage renal disease was highest in the high tertile group among the three tertile groups. For the cohort without DM, no significant difference was observed in the cause of end-stage renal disease among the three tertile groups (*P* = 0.094). Differences were observed in the urine volume and ultrafiltration volume among three tertile groups; by contrast, no difference was observed in the total volume of water removed among three tertile groups. We observed no significant differences in proportions of sex or age, dialysis vintage, BMI, weekly Kt/V_urea_, CRP, proteinuria, calcium, phosphorus, sodium, potassium or nPNA levels among the tertiles.

**Table 1:** Patients' clinical characteristics.

	**Low tertile (*n* = 66)**	**Middle tertile (*n* = 67)**	**High tertile (*n* = 66)**	***P*****-value**
Age (years)	54.5 ± 12.7	55.9 ± 12.0	56.3 ± 12.1	0.686
Sex (men)	33 (50.0%)	36 (53.7%)	44 (66.7%)	0.128
Diabetes mellitus (%)	26 (39.4%)	31 (46.3%)	41(62.1%)	0.028
Underlying disease of ESRD				0.031
Diabetes mellitus	24 (36.4%)	25 (37.3%)	36 (54.5%)	
Hypertension	15 (22.7%)	26 (38.8%)	11 (16.7%)	
Chronic glomerulonephritis	19 (28.8%)	9 (13.4%)	15 (22.7%)	
Others	6 (9.1%)	3 (4.5%)	2 (3.0%)	
Unknown	2 (3.0%)	4 (6.0%)	2 (3.0%)	
Automated peritoneal dialysis	30 (45.5%)	15 (22.4%)	12 (18.2%)	0.001
Dialysis vintage (months)	38 (58)	51 (71)	62 (75)	0.151
Body mass index (kg/m^2^)	24.3 ± 4.0	24.3 ± 3.2	25.2 ± 4.0	0.381
Weekly Kt/V_urea_	1.92 ± 0.45	1.96 ± 0.50	1.87 ± 0.43	0.521
C-reactive protein (mg/dL)	0.15 (0.41)	0.16 (0.35)	0.18 (0.55)	0.669
DP4_Cr_	0.62 ± 0.14	0.66 ± 0.13	0.69 ± 0.12^*^	0.006
Urine volume (ml/day)	285 (755)	200 (864)	0 (463)^*^	0.013
Ultrafiltration volume (ml/day)	781 (784)	950 (618)	1186 (913)^*^	0.002
Total volume of water removed (ml/day)	1273 (791)	1322 (840)	1412 (778)	0.296
Proteinuria (mg/day)	201 (569)	93 (553)	0 (173)	0.060
Edema index	0.397 ± 0.015	0.398 ± 0.011	0.405 ± 0.012^*^^^#^^	0.001
Serum calcium (mg/dL)	8.4 ± 0.9	8.3 ± 0.9	8.1 ± 1.0	0.302
Serum phosphorus (mg/dL)	4.9 (1.9)	4.9 (2.0)	4.9 (1.9)	0.302
Serum sodium (mEq/L)	140 ± 2	140 ± 4	136 ± 4	0.081
Serum potassium (mEq/L)	4.4 ± 0.7	4.5 ± 0.7	4.7 ± 0.7	0.148
Serum albumin (g/dL)	3.7 ± 0.5	3.6 ± 0.4	3.4 ± 0.5^*^	0.001
nPNA (g/kg/day)	0.83 ± 0.20	0.81 ± 0.19	0.87 ± 0.23	0.263

*Data are expressed as mean ± standard deviation for continuous variables with normal distribution or median (interquartile range) for continuous variables with non-normal distribution and as number (percentage) for categorical variables. P-values were tested using one-way analysis of variance for variables with normal distribution or Kruskal-Wallis test for variables with non-normal distribution, followed by a Bonferroni's post-hoc comparison for continuous variables and Pearson's χ^2^ or Fisher's exact tests for categorical variables*.

*ESRD, end-stage renal disease; DP4_Cr_, 4-h dialysate-to-plasma creatinine concentration ratio; nPNA, normalized protein equivalent of total nitrogen appearance*.

* *P < 0.05 compared with low tertile and*

# *P < 0.05 compared with middle tertile*.

### Association Between PPL and ALM Index or HGS

HGS in the low, middle, and high tertiles was 23.4 ± 9.2, 23.8 ± 8.9, and 23.6 ± 8.3 kg, respectively (*P* = 0.967). The ALM index in the low, middle, and high tertiles was 6.0 ± 1.3, 6.0 ± 1.2, and 6.5 ± 1.1 kg/m^2^, respectively (*P* = 0.061). Multivariate analysis showed that the HGS values in the low, middle, and high tertile groups were 22.7 ± 0.8, 23.8 ± 0.7, and 24.2 ± 0.8 kg, respectively (*P* = 0.384), while the ALM index values were 6.1 ± 0.1, 6.1 ± 0.1, and 6.4 ± 0.1 kg/m^2^, respectively (*P* = 0.146). The proportions of patients with low muscle mass in the low, middle, and high tertiles was 43 (65.2%), 45 (67.2%), and 34 (51.5%), respectively (*P* = 0.132). The proportions of patients with low HGS in the low, middle, and high tertiles was 31 (47.0%), 28 (41.8%), and 36 (54.5%), respectively (*P* = 0.334). The proportions of patients with sarcopenia in the low, middle, and high tertiles was 24 (36.4%), 19 (28.4%), and 21 (31.8%), respectively (*P* = 0.612). Logistic regression analysis showed that the odds ratios for sarcopenia with an increasing tertile were 0.90 (95% CI, 0.63–1.30; *P* = 0.576) in the univariate analysis and 0.70 (95% CI, 0.44–1.11; *P* = 0.126) in the multivariate analysis.

Linear regression analysis showed that PPL was not associated with HGS or ALM index (standardized β ± standard error and *P*-value was −0.032 ± 0.000 and 0.650 for HGS and 0.020 ± 0.000 and 0.781 for ALM index). Multivariate linear regression analysis showed that the standardized β ± standard errors of PPL were 0.117 ± 1.060 for HGS (*P* = 0.185) and 0.089 ± 0.192 for ALM index (*P* = 0.441), respectively. Correlation coefficients with PPL were −0.032 for HGS, 0.020 for ALM index, 0.157 for nPNA, −0.343 for serum albumin, 0.276 for edema index, and 0.220 for DP4_Cr_ (*P*-values were 0.650 for HGS, 0.781 for ALM index, 0.030 for nPNA, <0.001 for serum albumin, <0.001 for edema index, and 0.002 for DP4_Cr_). Subgroup analyses for age, sex, DM, or dialysis modality showed similar trends as those from total patients (Table 2).

**Table 2:** Correlation between peritoneal protein loss and various indices by subgroups.

	**Age**				**Sex**			
	**<65 years (*****n*** **= 153)**		**≥65 years (*****n*** **= 46)**		**Men (*****n*** **= 113)**		**Women (*****n*** **= 86)**	
	***r***	***P*****-value**	***r***	***P*****-value**	***r***	***P*****-value**	***r***	***P*****-value**
Handgrip strength (kg)	−0.033	0.690	0.022	0.882	−0.148	0.118	0.028	0.796
ALM index (kg/m^2^)	0.019	0.820	0.078	0.605	0.003	0.974	0.006	0.960
nPNA (g/kg/day)	0.157	0.057	0.159	0.290	0.093	0.336	0.241	0.027
Serum albumin (g/dL)	−0.353	<0.001	−0.308	0.038	−0.351	<0.001	−0.339	0.001
Edema index	0.336	<0.001	0.048	0.750	0.216	0.022	0.398	<0.001
DP4_Cr_	0.223	0.006	0.222	0.138	0.317	0.001	0.105	0.337
	**DM**				**Dialysis modality**			
	**Non-DM (*****n*** **= 101)**		**DM (*****n*** **= 98)**		**CAPD (*****n*** **= 142)**		**APD (*****n*** **= 57)**	
	***r***	***P*****-value**	***r***	***P*****-value**	***r***	***P*****-value**	***r***	***P*****-value**
Handgrip strength (kg)	−0.091	0.365	0.027	0.795	−0.067	0.426	0.162	0.228
ALM index (kg/m^2^)	−0.065	0.517	0.145	0.156	0.033	0.696	0.064	0.634
nPNA (g/kg/day)	0.287	0.004	0.104	0.314	0.115	0.176	0.253	0.065
Serum albumin (g/dL)	−0.396	<0.001	−0.274	0.006	−0.391	<0.001	−0.183	0.172
Edema index	0.312	0.002	0.162	0.112	0.222	0.008	0.374	0.004
DP4_Cr_	0.299	0.002	0.110	0.281	0.250	0.003	0.227	0.089

*Correlation analyses were analyzed using Pearson's correlation*.

*r, correlation coefficient; ALM, appendicular lean mass; nPNA, normalized protein equivalent of total nitrogen appearance; DP4_Cr_, 4-h dialysate-to-plasma creatinine concentration ratio; DM, diabetes mellitus; CAPD, continuous ambulatory peritoneal dialysis; APD, automated peritoneal dialysis*.

## Discussion

In our study, we evaluated the association between PPL and HGS or ALM index as two key indicators for sarcopenia. First, we evaluated differences in HGS and ALM index according to tertiles of PPL. Univariate analyses did not reveal significant differences among tertiles. Second, PPL was not associated with HGS and ALM index on correlation and linear regression analyses. Third, subgroup analyses did not reveal any association as well. In our study, there were significant differences in prevalence of DM, dialysis modality, DP4_Cr_, edema index, serum albumin, or urine volume among the three tertile groups. These factors may influence sarcopenia associated factors. However, multivariate analysis is useful to adjust for covariates associated with the outcome measures and predict the independent effect of specific variables. Similar results were obtained from those using multivariate analyses.

Our study showed that, in the aspect of sarcopenia, patients with high PPL were not inferior to those with low PPL, and this finding may be an extension of the middle molecule hypothesis (21). Since the introduction of CAPD as a portable or wearable form of PD by Popovich et al., many investigations regarding solute/water removal during PD have been performed (22). Previous studies identified that hemodialysis (HD), based on markedly different Kt/V_urea_ between PD and HD, has superior clearance of urea as a small uremic toxin compared with PD, but the prevalence of uremic complications such as neuropathy was lower in PD patients than in HD patients (21, 23). They introduced the middle molecule hypothesis, which explains the favorable removal of middle molecules during PD. Middle molecules, which weigh around 0.5–60 kD, are more effectively removed by convection combined with water removal than diffusion, which is dependent upon the difference in the molecule's concentration, and are removed in a time-dependent manner (24–26). The peritoneal membrane is more porous than the HD membrane, and PD has longer dialysis time (to 24 h of a day and 7 days a week) than intermittent HD. These two factors are associated with favorable removal of middle molecules (25). Various factors, associated with middle molecule removal such as the proportions of large size pore or vascularity within the peritoneal membrane, are associated with PPL among PD patients. The middle molecules are more effectively removed in patients with high PPL than in those with low PPL.

This favorable removal of middle molecules coincided with the removal of large amount of albumin as a nutrient. However, the clinical impact of albumin removal due to PPL differs from that of albumin deficiency as malnutrition. Protein-bound uremic toxins, such as indoxyl sulfate, were not effectively removed by conventional dialysis and were associated with accumulation of these toxins, resulting in various complications. In addition, an uremic condition may promote the carbamylation of serum albumin, and the modified albumin can cause harmful effects, such as oxidative stress, erythropoietin resistance, uremic symptoms or mortality (27–31). These findings suggest that compensable albumin loss during dialysis can be associated with favorable outcomes. A previous study showed that patients with high albumin loss during HD had better outcomes than those with low albumin loss (13). This paradoxical association was confirmed based on the results of comparative studies on HD and hemodiafiltration (HDF). A previous study compared the sarcopenia-associated factors between high-volume HDF and high-flux HD, and showed that patients who underwent high-volume HDF exhibited a trend of lower serum albumin level than those who underwent high-flux HD. However, patients who underwent high-volume HDF had higher muscle mass and protein intake than those who underwent high-flux HD (14). These results suggest that if the patient does not have liver disease, significant inflammation status, or severe malnutrition, loss of acceptable amounts of albumin during dialysis may be associated with favorable outcomes via removal of protein-bound uremic toxin or unhealthy albumin. In our study, patients with high PPL had poor urine volume and high proportions of DM, increasing their risk of developing sarcopenia. However, loss of acceptable amounts of albumin may reduce the risk of developing sarcopenia.

PPL positively correlated with DP4_Cr_. Therefore, in most studies regarding the association between PPL and nutritional status, DP4_Cr_ as an indirect indicator for PPL would be used rather than PPL. Harty et al. evaluated the association of DP4_Cr_ or PPL with serum albumin in 147 PD patients (15). They found that both DP4_Cr_ and PPL were inversely associated with serum albumin level, but the PPL alone was not independently associated with serum albumin. In addition, lean mass was evaluated by creatinine kinetics, and no association was observed between DP4_Cr_ and lean mass. Kang et al. evaluated the association between peritoneal permeability using DP4_Cr_ and various nutritional indicators in 147 PD patients (16). They showed that high transporter was associated with low serum albumin, insulin-like growth factor-1, and lean mass using creatinine kinetics, but not through bioimpedance. Szeto et al. evaluated nutritional status according to peritoneal transport status using DP4_Cr_ (17). They evaluated changes in lean mass using creatinine kinetics and serum albumin for 2 years, and baseline DP4_Cr_ values were found to be inversely associated with serum albumin, nPNA, and lean mass. However, there was no significant difference in longitudinal changes by peritoneal transport status. Chung et al. evaluated albumin, nPNA, lean mass using creatinine kinetics according peritoneal transport status using DP4Cr and showed inverse association between peritoneal transporter status and nutritional index (18).

We divided our patients into three tertiles according to PPL regarding previous two studies and evaluated HGS and ALM index using DXA as the final outcome of malnutrition in dialysis patients (10, 12). Both, PPL tertile as a categorical variable and PPL as a continuous variable were not associated with ALM index. Laboratory markers such as serum albumin or creatinine were well-known nutrition indices, but these can be largely influenced by non-nutritional status such as volume overload or inflammation. Our study also showed an inverse correlation between edema index and serum albumin (*r* = −0.499, *P* < 0.001), and PPL also inversely correlated with serum albumin through increase in edema index (*r* = −0.343, *P* < 0.001). Muscle mass is useful to reflect the chronic change of malnutrition as it can help exclude acute changes caused by volume or inflammation. We evaluated lean mass using DXA, which is considered as the reference method for predicting muscle mass (32). DXA is also influenced by hydration status. However, in our study, mean edema index was 0.397 for the low tertile group and 0.405 for high tertile group, and the difference in mean edema index between the low and high tertiles was approximately 2%. The effect of lean mass overestimation by volume may be attenuated by small differences in edema index. The correlation between edema index and ALM index was not significant (*r* = −0.048, *P* = 0.499). These reveal that the effect of volume was less in the ALM index than in laboratory markers.

Decrease in muscle strength can develop earlier than decrease in muscle mass (33). In addition, muscle strength measurements may be the accurate method for predicting muscle mass rather than directly measuring muscle mass in patients who are influenced by volume status. Our study evaluated HGS as a volume-independent method, and there was no significant difference among PPL tertiles. Finally, sarcopenia as a merged category using muscle mass and strength was not associated with PPL. Subgroup analyses using age, sex, the presence of DM, or dialysis modality showed similar trends. However, nPNA and serum albumin were associated with PPL. In our study, nPNA positively correlated with PPL, probably caused by increase in protein catabolism through high PPL. In addition, serum albumin was inversely correlated with PPL associated with hypervolemia in patients with high PPL. This reveals that laboratory markers such as nPNA or serum albumin were associated with PPL *via* non-nutritional status such as volume, but PPL is not associated with muscle mass or strength as a long-term marker of malnutrition. These results coincide with previous opinions showing that PPL can be compensated by adequate dietary intake (34). All patients in our study were followed up in the outpatient department and were relatively stable, leading to a conclusion of non-association between PPL and sarcopenic indicators.

In our study, the high tertile group had higher prevalence of DM and proportion of CAPD than the other tertile groups. This association may be an interesting issue in PD patients. Fast peritoneal transport is associated with increase in peritoneal vascularity and inflammation or vascular injury (3, 8, 35, 36). PPL is more closely associated with increased numbers of large pores by local inflammation than peritoneal vascularity *per se* (35). Although a small solute transport does not completely coincide with PPL, the DP4_Cr_ in DM patients is higher than that in non-DM patients and is positively correlated with PPL. These associations may explain the high prevalence of DM in patients with high PPL. No difference was found in PPL according to dialysis modality from previous studies (10, 37, 38). Dialysis modality may not be directly associated with PPL, but the difference in dialysis modality according to PPL may be due to the differences in residual renal function. Over half of the patients in the high tertile group developed anuria. The efficacy of sodium removal during APD is lower than that during CAPD, and patients with low residual renal function have a higher proportion of undergoing CAPD than APD (39, 40). It may be associated with the higher proportion of CAPD in the high tertile group than the other groups.

Our study has inherent limitations. It was a single-center and retrospective cross-sectional study. Therefore, causality between PPL and sarcopenia could not be identified. Second, DXA is commonly used to predict muscle mass, but the effect of volume cannot completely be excluded. Third, our study did not include data on physical performance, which could be more accurate in predicting muscle function than muscle mass or strength. Fourth, our study did not include data on serum or dialysate levels of middle molecules or protein-bound uremic toxins, such as β2-microglobulin or indoxyl sulfate. In our study, the non-association between PPL and sarcopenic components may be due to theoretical removal of these molecules. Moreover, we were unable to confirm the causal relationships among PPL, uremic toxin removal, and sarcopenic components.

The present study showed that PPL is not independently associated with muscle mass, strength, and sarcopenia in PD patients. However, considering the methodological limitations, prospective longitudinal studies, including volume-independent muscle measurements and physical performance, and a large number would be ideal to identify the association between PPL and sarcopenia in PD patients.

## Data Availability Statement

The raw data supporting the conclusions of this article will be made available by the authors, without undue reservation.

## Ethics Statement

The studies involving human participants were reviewed and approved by the Institutional Review Board of the Yeungnam University Medical Center (Approval No: 2021-01-033). Written informed consent for participation was not required for this study in accordance with the national legislation and the institutional requirements.

## Author Contributions

SK: conceptualization, methodology, formal analysis, and investigation. AK: software and data curation. JD and SK: validation, writing—review and editing. JD: resources, supervision, project administration, and funding acquisition. SK and AK: writing—original draft preparation. All authors have read and agreed with the published version of the manuscript.

## Conflict of Interest

The authors declare that the research was conducted in the absence of any commercial or financial relationships that could be construed as a potential conflict of interest.
